# Lipidomic response of the entomopathogenic fungus *Beauveria bassiana* to pyrethroids

**DOI:** 10.1038/s41598-021-00702-y

**Published:** 2021-10-29

**Authors:** Anna Litwin, Przemysław Bernat, Monika Nowak, Mirosława Słaba, Sylwia Różalska

**Affiliations:** Department of Industrial Microbiology and Biotechnology, Institute of Microbiology, Biotechnology and Immunology, Faculty of Biology and Environmental Protection, University of Lodz, Lodz, Poland

**Keywords:** Biotechnology, Microbiology, Environmental sciences

## Abstract

Pyrethroids are chemical insecticides that are widely used to control pests. Entomopathogenic fungi are considered environmentally safe alternatives to these compounds. Pyrethroids and entomopathogenic fungi not only co-exist in the environment but can also be applied together in pest control. They are often found in contact with each other, and thus, it seems important to understand their interactions at the cellular level. In this study, we analyzed whether pyrethroids could influence the phospholipid profile of *Beauveria bassiana* and whether membrane changes are one of the mechanisms by which these fungi adapt to unfavorable environmental conditions. The results of our study revealed that pyrethroids changed the phospholipid profile and increased the cell membrane permeability of *B. bassiana*, which enabled them to enter and accumulate within the fungal cells, resulting in oxidative stress. Pyrethroids influenced the amount of neutral lipids, caused a decrease in sodium content, and also temporarily lowered the level of the secondary metabolite oosporein in the studied fungi. These findings indicate that the effect of pyrethroids on entomopathogenic fungi may be more complex than originally thought and that lipidomic studies can aid in fully understanding the influence of these chemicals on the mentioned group of fungi.

## Introduction

Pyrethroids are insecticides used to control arthropods in agriculture, forestry, and greenhouses. They are neurotoxic substances that disrupt the nervous system of insects by affecting the activity of sodium and chloride channels^1–3^. Due to the widespread use as well as the lipophilic and hydrophobic properties of pyrethroids, their residues can be found in the environment (e.g., soil, surface water, and sediments)^2,4^. Despite the fact that pyrethroids are less harmful and safer than other insecticides, it has been proven that they exert negative effects on living organisms^4^. Pyrethroids have rather long half-lives in soil and are also poorly soluble in water due to their strongly hydrophobic nature^2^.

The soil is the habitat of many microorganisms, including entomopathogenic fungi, which are parasites controlling the density of insect populations in nature. It has been estimated that the propagules of *Ascomycota* entomopathogenic fungi could reach 10^6^ per g of soil^5^. Entomopathogens of the genera *Beauveria* and *Metarhizium* are widely used as bioinsecticides and introduced into the environment to protect crops^6^. Due to their long-term action, these fungi can be used together with chemical insecticides or as their alternatives in pest control^7^.

Entomopathogenic fungi and pyrethroids can not only be applied together to control pests but also coexist in the environment, and are therefore often found in contact with each other. Thus, it seems important to understand their interactions at the cellular level.

So far, only a few studies have analyzed the response of entomopathogenic fungi to pyrethroids at the cellular level, with most of them focusing only on fungal growth in the presence of these compounds^8–11^. Pyrethroids do not mostly adversely affect fungal growth^12^, and hence are considered to not cause any significant cell damage. Forlani et al.^8^ revealed that exposure of *Beauveria bassiana* to the pyrethroid deltamethrin led to the induction of two cytochrome P450 genes and antioxidant system genes such as superoxide dismutase, catalase, and glutathione S-transferase. These findings suggest that the defense mechanisms of *B. bassiana* are activated in response to the harmful effects of deltamethrin.

In this study, we analyzed whether pyrethroids, which are considered nontoxic to entomopathogenic fungi, influenced the phospholipid profile of *B. bassiana* due to the fact that changes in the membrane structure and properties are one of the mechanisms that allow microorganisms to adapt to unfavorable environmental conditions and that even slight changes in lipidome may affect the functioning of *B. bassiana* fungi in the natural environment.

## Materials and methods

### Reagents

The insecticides used in the study were λ-cyhalothrin, α-cypermethrin, and deltamethrin. Stock solutions of pyrethroids were prepared in 10 mg mL^−1^ acetonitrile. The other reagents used were phosphate-buffered saline (PBS), propidium iodide, ammonium formate, formic acid, cholesterol, BSTFA/TMCS solution (N,O-bis(trimethylsilyl)trifluoroacetamide/trimethylchlorosilane), DAB (3,3′-diaminobenzidine tetrahydrochloride hydrate), NBT (Nitrotetrazolium Blue chloride), sodium dihydrogen phosphate and disodium hydrogen phosphate dodecahydrate, chloroform, methanol, methyl tert-butyl ether, isopropyl alcohol, methanolic KOH, HCl, and nitric acid.

### Strain and growth conditions

The strain *Beauveria bassiana* ARSEF 2860 used in the study was obtained from the ARSEF (Agricultural Research Service Collection of Entomopathogenic Fungal Cultures, USA). It was isolated from *Schizaphis graminum* and can be potentially applied in the biological control of aphids and spider mites^13,14^. Moreover, its genome has been sequenced and is therefore commonly used in scientific research^15–17^. For the experiments, conidia were harvested from 14-day-old cultures of *B. bassiana* grown on Sabouraud dextrose agar slant and transferred to 40 mL Sabouraud dextrose broth medium at a density of 10^6^ per mL. The medium was supplemented with the abovementioned insecticides at final concentrations of 5, 50, and 100 mg L^−1^. The inoculated medium was incubated in a rotary shaker (120 rpm) for 24, 36, and 48 h at 28 °C.

### Dry mass measurements, metabolic activity analysis, and microscopy

The effect of pyrethroids on the growth of *B. bassiana* was determined by measuring the dry mass of cultures. After incubation for 36 and 48 h, the cultures were filtered under reduced pressure through a Whatman membrane filter (0.45 μm) and dried at 100 °C for 1 h to achieve a constant weight. The effect of pyrethroids on the viability (metabolic activity) of *B. bassiana* was determined by a method using fluorescein diacetate (FDA) with additional modifications^18^. Briefly, the cultures were filtered using a vacuum pump, and the entire wet mass from the flask was divided into portions and transferred to the wells of a 24-well plate. To each well, 1 mL of FDA in sodium phosphate buffer (pH 6.4, 0.7 mg mL^−1^) was added. The samples were incubated in the dark at 28 °C for 15 min, and the fluorescence of the wells was then read using a plate reader at an excitation wavelength of 485 nm and an emission wavelength of 530 nm. The fluorescence values were divided by the amount of dry mass estimated for each sample. The results were expressed as % viability in comparison to the biotic control.

Microscopic observations were made on samples mounted on slides. Images were captured in a Nikon Eclipse E200 microscope equipped with a digital camera and Nikon E Plan objective (40x/0.6) with DeltaPix InSight software.

### Determination of sodium, magnesium, potassium, and calcium

The effect of pyrethroids on the content of sodium, magnesium, potassium, and calcium of entomopathogenic fungi was determined in mycelium originating from the insecticide-exposed cultures. Briefly, mycelia were separated from cultures using a filter paper and dried at 100 °C to obtain dry weight. Subsequently, the biomass was mineralized with concentrated nitric acid (65%). The content of metals in the samples was estimated by atomic absorption spectrometry (AAS) using a Spectra 240 FS apparatus.

### Ergosterol measurement

To determine the content of ergosterol in fungal biomass, 100 mg of biomass was placed in an Eppendorf tube with glass beads. The samples were frozen in liquid nitrogen and then homogenized using a FastPrep-24 instrument for 20 s at 5 m s^−1^. Ergosterol was extracted as described by Stolarek et al.^19^ with additional modifications. Briefly, 1 mL of methyl tert-butyl ether–methanol mixture (3:1, v/v) was added to the samples and the samples were vortexed. Next, 650 μL of water–methanol mixture (3:1, v/v) was added, and the samples were vortexed again and centrifuged at 5000 × *g* for 5 min at 10 °C. After centrifugation, the top layer was collected and evaporated. Then, the samples were dissolved in a mixture of 0.5 mL of chloroform, 0.5 mL of methanolic KOH, and 20 μg of cholesterol (20 μL from stock solution at the concentration of 1 mg mL^−1^). After incubating the samples for 1 h at 23 °C, 0.325 mL of 1 M HCl and 0.125 mL of deionized water were added and centrifuged (5000 × g). Subsequently, the lower layer was transferred to a new Eppendorf tube and dried for 12 h under fume hood. Then, 100 µL of the BSTFA/TMCS solution was added and the samples were incubated for 90 min at 85 °C. After incubation, 50 µL of hexane was added and the samples were transferred to chromatographic tubes. The content of ergosterol in the samples was determined by gas chromatography–tandem mass spectrometry as described previously using an Agilent 7890 system equipped with an HP 5 MS column and a 5975C mass detector^20^.

### Phospholipid analysis

For analyzing the phospholipid profile, samples were extracted as described in the previous section. After evaporation, the extract was dissolved in 1 mL of methanol and analyzed by liquid chromatography–tandem mass spectrometry (LC–MS/MS) using an LC Agilent 1200 system coupled with a Sciex QTRAP 4500 tandem mass spectrometer. A Kinetex C18 column (50 mm × 2.1 mm, particle size 5 μm) heated to 40 °C with a flow rate of 500 μL min^−1^ was used for this purpose. The ion source of the mass spectrometer was operated in a negative mode under the following conditions: spray voltage 4.500 V, curtain gas 25, nebulizer gas 60, auxiliary gas 50, and temperature 600 °C.

### Membrane permeability assay

For determining membrane permeability, 1 mL of each culture was transferred to an Eppendorf tube and the samples were centrifuged. The supernatant was removed, and 1 mL of PBS and 2 μL of propidium iodide at a concentration of 0.1 mg mL^−1^ were added. Subsequently, the samples were incubated in the dark at room temperature for 5 min. After incubation, the mycelium was washed twice in PBS, suspended in 1 mL of PBS, and transferred to a 24-well titration plate. Fluorescence of the samples was measured using a FLUOstar Omega fluorescence microplate reader (excitation wavelength: 540 nm, emission wavelength: 610 nm), with the fluorescence of the supernatant set as a background. The results were expressed as a fluorescence unit (U) per mg of dry mass.

### Extraction and quantification of pyrethroids

For estimating the content of pyrethroids, the medium was separated from the biomass by filtration and extracted with ethyl acetate followed by methylene chloride. The amount of insecticides in the mycelium and culture medium was determined using a gas chromatography–mass spectrometry system equipped with an HP 5 MS column (30 m × 250 µm × 0.25 µm) and a 5975C mass detector.

### Quantification of neutral lipids

Triacylglicerols (TAGs) and diacylglycerols (DAGs) were extracted as described in "Ergosterol measurement" section. After evaporation, the samples were dissolved in 1 mL of methanol. The content of acylglycerols was determined by LC–MS/MS. To detect acylglycerol, ammonium adducts of multiple reaction monitoring (MRM) scans including parent–daughter pairs were used. Chromatographic separation was conducted on a C18 column heated to 40 °C, and detection was performed by single-ion monitoring and the enhanced product ion method. Water and a mixture of acetonitrile–isopropyl alcohol (5:2) containing 5 mM ammonium formate and 0.1% formic acid were used as mobile phases^19^.

### Oxidative stress

To determine the content of hydrogen peroxide, 1 mL of the culture was centrifuged at 2000 × *g* for 5 min. The obtained biomass was suspended in 1 mL of DAB solution (1 mg mL^−1^ of DAB was dissolved in sodium phosphate buffer (pH 7.8) and pH was adjusted to 3.8 before adding the solution to the biomass, and the samples were incubated in sunlight for 1 h.

The content of superoxide anion in *B. bassiana* hyphae was measured using the method described by Nykiel-Szymańska et al.^21^ Briefly, 1 mL of the culture was centrifuged at 2000 × *g* for 5 min. The obtained biomass was suspended in 1 mL of the solution containing 0.1% NBT and 10 mM sodium azide dissolved in sodium phosphate buffer (pH 7.8). The samples were incubated for 1 h in the dark at room temperature.

The content of hydrogen peroxide and superoxide anion in *B. bassiana* hyphae was examined under an LSM510 Meta confocal laser scanning microscope. The results were expressed as a percentage of the stained area compared to the total hyphal area.

### Oosporein determination

To determine the content of oosporein pigment, the cultures were centrifuged at 5000 × *g* for 15 min. Then, the medium was acidified to pH 2 with hydrochloric acid. The acidified medium was extracted twice with ethyl acetate for 3 min, and the obtained extracts were dehydrated with anhydrous sodium sulfate. Next, the extracts were filtered through a paper filter, and the filtrate was evaporated using a vacuum evaporator. Subsequently, the samples were dissolved in 1 mL of methanol and analyzed using an LC Agilent 1200 system coupled with a Sciex QTRAP 3200 tandem mass spectrometer.

Flow injection analysis of samples was conducted with an injection volume of 10 µL and a flow rate of 600 µL min^−1^. A mixture of water with 0.1% formic acid (solvent A) and a mixture of acetonitrile with 0.1% formic acid (solvent B) were used as mobile phases. A phase A–phase B ratio of 30:70 was maintained for 0.7 min. Tandem mass spectrometry detection was conducted using the electrospray ion source ESI with negative polarization. To confirm the presence of oosporein, the three following MRM pairs were determined: Q1/Q3—305.1/177.1, 305.1/149.1, and 305.1/133.1. Of these, the quantitative pair was 305.1/177.1 The individual analytes parameters were: entrance potential (EP; (-5)), collision cell entrance potential (CEP; (-30)), collision energy (CE; (-30)), collision cell exit potential (CXP; (-1)), declustering potential (DP (-35)). The amount of oosporein was determined based on a calibration curve over a concentration range of 1–100 µg, with quadratic regression, where the "*R*" value was 0.9954.

### Statistical analyses

All samples were prepared in triplicate. The experiments were repeated twice, and the standard deviation (SD) of the results was determined. The normality of the data distribution was determined by the Shapiro–Wilk test. Data were analyzed using one-way analysis of variance (ANOVA), and the means were compared using Tukey's post hoc test. A *p*-value of < 0.05 was considered statistically significant. Statistical analyses were conducted in the STATISTICA version 13.3 software (StatSoft).

## Results and discussion

### Effect of pyrethroids on *B. bassiana* dry biomass content and metabolic activity

After 36 h of incubation, statistically significant (*P* < 0.01) changes in biomass dry weight were observed in the *B. bassiana* samples supplemented with pyrethroids at the concentrations of 50 and 100 mg L^−1^ (Supplementary Table S1). However, after 48 h of incubation, statistically significant changes were noted only in the samples supplemented with 100 mg L^−1^of λ-cyhalothrin (*P* = 0.00169) and α-cypermethrin (*P* = 0.015635). The results of our study also indicated that the tested pyrethroids did not affect the growth of *B. bassiana* at the concentration of 5 mg L^−1^.

Intriguingly, microscopic observations did not reveal any morphological changes in mycelia exposed to pyrethroids (Supplementary Fig S1 and Supplementary Fig S2) but we noted a significant decrease in the number of blastospores incubated for 24 and 48 h with pesticides at the concentrations of 50 and 100 mg L^−1^ (Supplementary Table S2). Among the tested insecticides, cyhalothrin had the most detrimental effect on this process. According to our knowledge, there are no data regarding the effect of pyrethroids on blastospores formation in liquid cultures. However, in the cultures on solid media, a decrease in the sporulation of *B. bassiana* was noted in the presence of cypermethrin and deltamethrin^22^. Inhibition of *B. bassiana* sporulation by herbicides in solid media has also been documented^23^. It seems that xenobiotics quite often inhibit the conidiation process, but the molecular mechanisms of this phenomenon are still unknown.

The study analyzed a wide range of insecticides concentrations, which were selected based on manufacturers’ recommended doses for field applications. The lowest concentration tested in the study (5 mg L^−1^) is tenfold lower than that recommended for commercial preparations for application per 100 m^2^ of the area. The concentration 50 mg L^−1^ is within the recommended concentration range of cypermethrin and deltamethrin but exceeds that of cyhalothrin. The highest tested concentration (100 mg L^−1^) is twice as high as the recommended concentration of cyhalothrin and deltamethrin. Because cypermethrin is used at high doses in the field, this concentration does not exceed the range of commonly applied range of doses.

The data reported in the literature are in line with our results. Pyrethroids used in recommended (and higher) doses strongly inhibited entomopathogenic fungi during the initial period of growth, limiting the rate of colony formation by more than 40%, while at lower doses these insecticides had a minimal inhibitory effect^24^.

We also checked whether the highest tested insecticide concentration influenced the metabolic activity of entomopathogenic fungi as previous studies have only analyzed the effect of pyrethroids on their growth. The tests conducted in our study did not show any negative effect of pyrethroids on the metabolic activity of *B. bassiana*, and even a slight increase in activity was found in the samples exposed to the tested substances (Table 1). In 24-h cultures, a 1.47-fold increase in metabolic activity was noted in the presence of deltamethrin (*P* = 0.000176), 1.36-fold increase in the presence of λ-cyhalothrin (*P* = 0.000237), and 1.32-fold increase in the presence of α-cypermethrin (*P* = 0.000376). In 36-h cultures, a 1.1-fold increase in metabolic activity was observed in the presence of λ-cyhalothrin (*P* = 0.016223); however, in the presence of α-cypermethrin and deltamethrin, the metabolic activity in the samples was comparable to that observed in the control. In 48-h cultures, no differences in metabolic activity were found between the cultures exposed to pyrethroids and the biotic control.

**Table 1:** Metabolic activity (%) of *B. bassiana* after 24, 36, and 48 h of culture with λ-cyhalothrin, α-cypermethrin, and deltamethrin.

	Control	Pyrethroids (100 mg L^−1^)		
		λ-cyhalothrin	α-cypermethrin	Deltamethrin
24 h	100.00 ± 4.76	135.73 ± 6.58**	132.46 ± 19.00**	146.85 ± 12.33**
36 h	100.00 ± 3.85	111.11 ± 11.76*	88.76 ± 12.80	98.26 ± 20.45
48 h	100.00 ± 12.39	113.59 ± 27.11	119.37 ± 17.51	120.79 ± 15.00

Data are means ± SD; all samples were prepared in triplicate, and the experiments were repeated twice. Results were tested by one-way ANOVA; significance: **P < 0.01, *P < 0.05.

Despite the growth inhibition, the tested insecticides did not adversely affect the metabolic activity of *B. bassiana*. This finding is different from the previous observations because most of the toxic compounds studied so far have been observed to cause a simultaneous reduction in the growth and viability of microorganisms. Różalska et al.^18^ used the same method in their study to examine the metabolic activity of *Metarhizium robertsii,* another entomopathogenic fungal species. The authors found that the addition of both 4-*n*-nonylphenol and technical nonylphenol caused a statistically significant decrease in the metabolic activity of the tested strain.

### Effect of pyrethroids on the content of sodium, potassium, calcium, and magnesium in biomass

All tested insecticides caused a decrease in sodium content in the biomass of *B. bassiana* (Table 2). The addition of 100 mg L^−1^ of λ-cyhalothrin and 50 and 100 mg L^−1^ of deltamethrin resulted in a two-fold reduction in the amount of this element in the fungal cells. To our knowledge, our study is the first to describe this phenomenon for fungi. The insecticidal action of pyrethroids is based on their binding to sodium voltage-gated channels in the neuronal membranes of insects^25^. Interestingly, it has been postulated that genes coding sodium voltage-gated channels are not present in the fungal genomes^26^ and the role of other sodium-transporting channels in fungi is still being elucidated.

**Table 2:** Content of sodium, magnesium, potassium, and calcium in the *B. bassiana* cells after cultivation with λ-cyhalothrin, α-cypermethrin, and deltamethrin.

Element mg g^-1^ dry mass	Control	Pyrethroids (mg L^-1^)								
		λ-cyhalothrin			α-cypermethrin			Deltamethrin		
		5	50	100	5	50	100	5	50	100
Na	2.68 ± 0.11	2.54 ± 0.18	1.84 ± 0.18**	1.31 ± 0.10**	1.94 ± 0.24**	1.64 ± 0.02**	1.73 ± 0.03**	1.50 ± 0.18**	1.39 ± 0.27**	1.26 ± 0.22**
Mg	0.28 ± 0.02	0.19 ± 0.04**	0.27 ± 0.03	0.34 ± 0.03*	0.18 ± 0.01**	0.22 ± 0.00*	0.22 ± 0.01*	0.31 ± 0.00	0.21 ± 0.00**	0.26 ± 0.02
K	11.28 ± 0.03	11.71 ± 0.52	8.77 ± 0.53**	7.43 ± 0.25**	8.47 ± 0.37**	8.64 ± 0.55**	10.64 ± 0.91	11.32 ± 0.24	9.13 ± 0.06**	8.79 ± 0.61**
Ca	2.46 ± 0.06	2.45 ± 0.09	2.06 ± 0.10**	2.25 ± 0.14	2.15 ± 0.01**	2.12 ± 0.14**	2.26 ± 0.09	2.16 ± 0.04**	1.90 ± 0.11**	1.68 ± 0.15**

Data are means ± SD; all samples were prepared in triplicate, and the experiments were repeated twice. Results were tested by one-way ANOVA; significance: **P < 0.01, *P < 0.05.

The presence of pyrethroids was also found to influence the content of other elements in *B. bassiana* biomass (Table 2). Cypermethrin (even at low concentration) decreased the levels of K, Mg, and Ca, while deltamethrin caused a decrease in their levels in fungal cells at a concentration of 50 mg L^−1^. Changes in the endogenous level of these elements might have resulted from increased membrane permeability after the exposure of fungal cells to pyrethroids.

A similar effect or leakage of potassium ions from the cells of *Candida albicans* associated with an increase in membrane permeability was observed by Lee and Lee (2014)^27^ after the treatment of cells with curcumin. The results of their study indicated that pyrethroids cause disturbances in the ionic balance not only in insects, but also in entomopathogenic fungi*.* On the other hand, our results showed that pyrethroids did not affect the viability of *B. basiana.* Therefore, further research is needed to elucidate the mechanism that enables the fungi to remain viable in the presence of pyrethroids, despite the negative impact of these compounds on the integrity of the cell membrane.

### Effect of pyrethroids on the ergosterol content in entomopathogenic fungi

The changes in the elemental content in fungal biomass may be associated with changes in the permeability of cell membranes. Ergosterol is an important marker of cell response to toxic substances. It plays a key role in many cell processes and is crucial for the integrity and functioning of the plasma membrane^20^.

**Figure 1:**
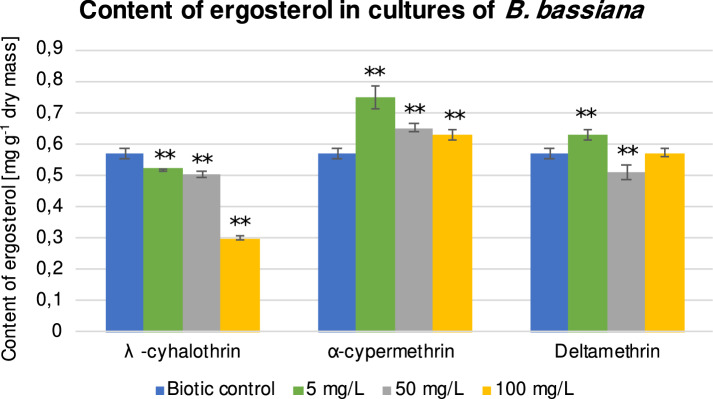
Content of ergosterol [mg g^−1^ dry mass] in the *B. bassiana* cells after 48 h of cultivation with λ-cyhalothrin, α-cypermethrin, and deltamethrin. All samples were prepared in triplicate, and the experiments were repeated twice. Results were tested by one-way ANOVA; significance: **P < 0.01, *P < 0.05.

Our study showed that the studied pyrethroids differed in their effects on the ergosterol content of *B. bassiana* cells. λ-cyhalothrin, at all tested concentrations (5, 50, and 100 mg L^−1^), caused a statistically significant decrease in content (by 8.78% (*P* = 0.000190), 12.29% (*P* = 0.000163), and 47.37% (*P* = 0.000163), respectively), in comparison to the biotic control), while α-cypermethrin caused an increase in its synthesis in *B. bassiana* cells (Fig. 1). In the case of deltamethrin, an increase in the ergosterol amount was observed at the concentration of 5 mg L^−1^, but at 50 mg L^−1^ a slight reduction in this component was observed in the fungal cells.

The literature has no information regarding the modifications in ergosterol level in the presence of pyrethroids in organisms, but the results observed in the content of elements and ergosterol in this work suggest that pyrethroids affect the cell membranes of *B. bassiana*.

Among the examined pyrethroids, the strongest membrane permeability in *B. bassiana* cells was found in presence of λ-cyhalothrin. Moreover, the lowest levels of ergosterol and higher phosphatidylcholine (PC)/phosphatidylethanolamine (PE) ratio were noticed in the cells in the presence of this insecticide. Considering the fact that ergosterol, PC, and PE regulate membrane permeability and fluidity, it seems that a decrease in the levels of ergosterol and PE caused an increase in the membrane permeability of fungal cells.

### Effect of pyrethroids on phospholipid compositions

The most important phospholipid classes determined in the *B. bassiana* membranes are presented in Table 3. Quantitative lipidomic analyses revealed the highest content of PC (51.08%) and PE (45.48%) in the membranes. The contents of phosphatidylinositol (PI), phosphatidylserine (PS), and phosphatidic acid (PA) was estimated as 2.78%, 0.58%, and 0.08%, respectively. In a previous study that examined the phospholipid profile of *B. bassiana,* PC was identified as the dominant class of phospholipids, followed by PE, the amount of which was found to be half of that of PC^28^. Other phospholipid classes (PS, PI, and PA) were also detected, with the content of PA being the lowest, as was also observed in our work.

**Table 3:** Phospholipid composition and PC/PE and PI/PS ratios determined in the *B. bassiana* cells after 48 h of cultivation with λ-cyhalothrin, α-cypermethrin, and deltamethrin.

Phospholipid class	Control	Pyrethroids (100 mg L^-1^)		
		λ-cyhalothrin	α-cypermethrin	Deltamethrin
PA relative abundance (%)	0.08 ± 0.03	0.06 ± 0.01	0.09 ± 0.03	0.07 ± 0.01
PC relative abundance (%)	51.08 ± 1.35	54.66 ± 0.37**	53.38 ± 0.92**	53.76 ± 0.57**
PE relative abundance (%)	45.48 ± 1.34	40.83 ± 0.74**	42.18 ± 0.94**	42.20 ± 0.51**
PI relative abundance (%)	2.78 ± 0.07	3.85 ± 0.48**	3.68 ± 0.32**	3.49 ± 0.19**
PS relative abundance (%)	0.58 ± 0.08	0.60 ± 0.10	0.66 ± 0.05	0.49 ± 0.09
PC/PE	1.12 ± 0.06	1.34 ± 0.03**	1.27 ± 0.05**	1.27 ± 0.03**
PI/PS	4.84 ± 0.83	6.58 ± 1.90	5.59 ± 0.89	7.37 ± 1.95*

Data are means ± SD; all samples were prepared in triplicate, and the experiments were repeated twice. Results were tested by one-way ANOVA; significance: **P < 0.01, *P < 0.05.

The lipidomic data of this study revealed that all tested pyrethroids affected the phospholipid profile of *B. bassiana* and caused a statistically significant increase in the PC content with a simultaneous decrease in the PE content (Table 3). As PC and PE are the main phospholipids in *B. bassiana* cells, the membrane dynamics of the cells depends on their ratio. PC is responsible for stabilizing the membrane bilayer phase, and maintaining its structure and functionality, while PE forms nonbilayer hexagonal phases. Changes in the phospholipid profile thus affect the properties of cell membranes^19^. The PC/PE ratio is also an essential factor of membrane integrity^29^. In this study, all the tested insecticides significantly increased this ratio (*P* = 0.000175, *P* = 0.000201, *P* = 0.000186 in the presence of λ-cyhalothrin, α-cypermethrin, and deltamethrin, respectively), thereby leading to higher membrane fluidity. In addition, the increased PC/PE ratios were in concordance with the changes observed in the endogenous levels of K, Mg, and Ca.

The LC–MS/MS analysis revealed that PC 18:2 18:2, PC 18:2 18:1, and PE 16:0 18:2 were the major lipids in *B. bassiana* cells and that pyrethroids had a noticeable influence on their content (Supplementary Table S3). A statistically significant increase in the level of unsaturated PC 18:2 18:2 was noted in the presence of λ-cyhalothrin (34.49 ± 0.64%, *P* = 0.000232), α-cypermethrin (33.85 ± 0.43%, *P* = 0.000238), and deltamethrin (33.15 ± 0.39%, *P* = 0.000289), compared to the biotic control (29.88 ± 0.25%). A higher amount of unsaturated PC shown by the analyses may also suggest an increase in membrane permeability. The level of PE 16:0 18:2, which was identified as the most predominant phospholipid, decreased to 18.04 ± 1.54% in the presence of λ-cyhalothrin (*P* = 0.005753), 18.62 ± 2.44% in the presence of α-cypermethrin (*P* = 0.024558), and 18.89 ± 1.30% in the presence of deltamethrin (*P* = 0.047449), compared to the biotic control (21.34 ± 1.11%).

All tested pyrethroids also caused a statistically significant increase (*P* < 0.001) in the PI content. The amount of PI 16:0 18:2 increased after the addition of λ-cyhalothrin (*P* = 0.005384), and that of PI 18:2 18:2 increased in the presence of all tested pyrethroids (*P* = 0.000178, *P* = 0.000181, *P* = 0.000302 with λ-cyhalothrin, α-cypermethrin, and deltamethrin, respectively) (Supplementary Table S3). PI acts as a precursor for the synthesis of many important lipid compounds, such as complex sphingolipids, phosphoinositides and inositol polyphosphates, and thus plays a key role in the basic metabolism. It is also essential for the anchoring of glycolipids in the plasma membrane^30^. Previous works have also demonstrated that toxic substances such as tributyltin caused an increase in PI content in fungal cell membranes^30^. No significant changes were observed in the content of PS (a precursor of PC and PE) and PA (a signal lipid) in this work.

### Effect of pyrethroids on acylglycerols content in entomopathogenic fungi

**Figure 2:**
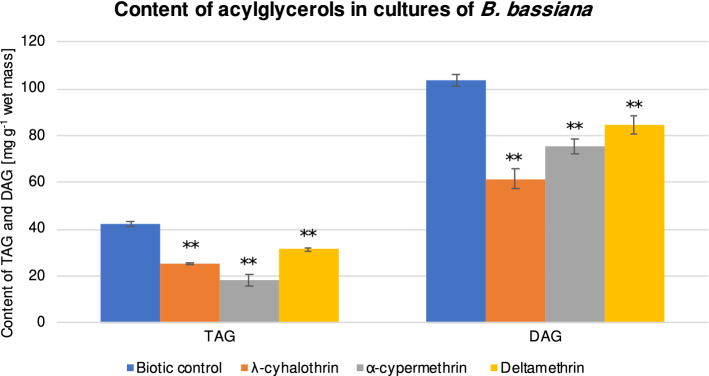
Content of tri- and diacylglycerols in the *B. bassiana* cells after 48 h of cultivation with λ-cyhalothrin, α-cypermethrin and deltamethrin at a concentration of 100 mg L^−1^. All samples were prepared in triplicate, and the experiments were repeated twice. Results were tested by one-way ANOVA; significance: **P < 0.01, *P < 0.05.

Neutral lipids, TAGs and DAGs, are the main reserve material and important element of the phospholipids biosynthesis pathway^31^. The present study revealed that pyrethroids reduced the total amount of acylglycerols and the content of DAGs was higher than that of TAGs in *B. bassiana* cells (Fig. 2). The most significant reduction in TAGs was observed in the samples exposed to α-cypermethrin, while λ-cyhalothrin caused the greatest decrease in the content of DAGs. It has been found that the precursor of acylglycerols in the TAG biosynthesis pathway is glycerol-3-phosphosphate, which is converted to lysophosphatidic acid and subsequently to phosphatidic acid. In the next steps, phosphatidic acid is converted to DAG, and then DAG is converted to TAG^32^. No data can be found in the literature regarding the inhibition of acylglycerol synthesis by pyrethroids in entomopathogenic fungi. Moreover, in *Donax trunculus* clam, a reverse effect has been observed after the exposure of the organisms to pyrethroids^33^.

Using LC–MS/MS, 13 TAG and 9 DAG species were identified in the *B. bassiana* samples (Supplementary Table S4). The major molecular TAGs detected were: 52:2, 54:3, 54:4, and 54:6 and DAGs were 34:2, 36:1, and 36:2. In the presence of pyrethroids, some changes in the content of individual neutral lipids were recorded. λ-Cyhalothrin caused a statistically significant decrease in the content of TAGs 52:4 (*P* = 0.041826), 54:1 (*P* = 0.000199), 54:2 (*P* = 0.000199), 54:3 (*P* = 0.002386), 54:4 (*P* = 0.000203), and 54:6 (*P* = 0.000338) and an increase in TAGs 48:0 (*P* = 0.000199), 50:0 (*P* = 0.000199), 52:1 (*P* = 0.000199), and 52:2 (*P* = 0.000239). α-Cypermethrin caused a statistically significant decrease in the content of TAGs 52:3 (*P* = 0.000417), 52:4 (*P* = 0.018714), 54:1 (*P* = 0.000199), 54:2 (*P* = 0.000199), 54:3 (*P* = 0.000250), 54:4 (*P* = 0.002145), and 54:6 (*P* = 0.000241) with a simultaneous increase in the content of TAGs 48:0 (*P* = 0.000199), 50:0 (*P* = 0.000199), and 52:1 (*P* = 0.000199). Our results indicate that deltamethrin had the weakest effect on individual TAG species and some changes were only recorded in the content of TAGs 54:0 (*P* = 0.000270) and 54:2 (*P* = 0.000199) and DAG 36:1 (*P* = 0.033229).

λ-Cyhalothrin and α-cypermethrin increased the amounts of DAGs 32:0 (*P* = 0.000199; *P* = 0.009261), and 34:2 (*P* = 0.000278; *P* = 0.000199); however, a higher content of DAG 34:0 (*P* = 0.005059) was also noticed in the *B. bassiana* samples cultivated with λ-cyhalothrin. Interestingly, the abundance of DAG 36:1 was found to be clearly reduced in the presence of λ-cyhalothrin (*P* = 0.000210) and α-cypermethrin (*P* = 0.000254), while deltamethrin caused an increase in the content of this acylglycerol (*P* = 0.033229).

Our results suggest that λ-cyhalothrin and α-cypermethrin increased the content of saturated TAGs and DAGs and decreased the content of unsaturated TAGs. Deltamethrin had a slight effect on the amounts of individual TAGs and DAGs.

Compared to the control, the lowest TAG/DAG ratio was found in the presence of cypermethrin (*P* = 0.003244), which may indicate increased accumulation of DAG related to TAG inhibition (Supplementary Table S4).

TAGs are the basic reserve material for cells and together with DAG they constitute an important element of phospholipid biosynthesis^19,34^ and also play a crucial role in protecting cells against oxidative damage^35^. Studies in mammals showed that accumulation of TAGs in lipid droplets prevents oxidative stress in cells by limiting the generation of reactive oxygen species (ROS)^35^. On the other hand, in this study, the fungal cells exposed to pyrethroids had a lower accumulation of TAGs accompanied by an increased percentage of hyphae containing superoxide anion radicals and hydrogen peroxide.

TAGs are also involved in fungal virulence, supporting the penetration of fungi into the insect cuticle^29,34^. This suggests that pyrethroids affect the metabolism of fungi, which play an important ecological role in the environment, controlling the insect populations.

### Effect of pyrethroids on membrane permeability and their accumulation

The membrane permeability of *B. bassiana* cells exposed to pyrethroids was determined by intracellular accumulation of propidium iodide (Table 4). The results showed a statistically significant increase in red fluorescence in fungal biomass incubated in the presence of λ-cyhalothrin and α-cypermethrin at the concentration of 100 mg L^−1^ (by 23.5%, *P* = 0.000196 and 15.7%, *P* = 0.000328, respectively) and a slight increase in the cultures containing deltamethrin at the concentration of 100 mg L^−1^ (7%). A statistically significant increase in the PC/PE ratio was also confirmed (Table 3). Furthermore, a higher content of ergosterol was noted in the samples supplemented with α-cypermethrin and deltamethrin, which proved an increase in the fluidity of *B. bassiana* cell membranes (Fig. 1).

**Table 4:** Membrane permeability and accumulation of pyrethroids in the *B. bassiana* cells.

	Control	Pyrethroids (100 mg L^-1^)		
		λ-cyhalothrin	α-cypermethrin	Deltamethrin
Membrane permeability [%]	100.00 ± 5.77	123.51 ± 4.49**	115.69 ± 4.10*	107.00 ± 1.80
Recovery [%]				
Culture medium	–	1.93 ± 0.24	2.50 ± 0.12	1.66 ± 0.33
Mycelium	–	71.84 ± 1.12	70.16 ± 0.83	99.64 ± 0.74

Data are means ± SD; all samples were prepared in triplicate, and the experiments were repeated twice. Results were tested by one-way ANOVA; significance: **P < 0.01, *P < 0.05.

Previous studies have also reported the changes in the fungal membranes permeability caused by pesticides. Higher membrane permeability associated with an increase in the PC/PE ratio was described for several strains of *Trichoderma* spp. cultured with chloroacetanilide herbicides^21^. Similarly, increased membrane fluidity was observed for the herbicide degrader *Umbelopsis isabellina* in the presence of 2,4-D^36^. Increased membrane fluidity was found to facilitate the transport of toxic substances into the fungal cells, which causes the accumulation or even degradation of these substances^7^.

In this study, all tested pyrethroids were found to be accumulated in the mycelium of *B. bassiana*. The amounts of λ-cyhalothrin and α-cypermethrin in fungal biomass were estimated at 71.84% and 70.16%, respectively, while deltamethrin was accumulated at 99.64%. This suggests that increased membrane permeability promotes the penetration of pyrethroids into the mycellium.

### Effect of pyrethroids on ROS production

Literature data indicate that the mechanism of toxicity of insecticides could be associated with enhanced intracellular ROS production/antioxidant enzyme activity^8^. Therefore, in this study, we determined the intracellular level of superoxide anion radicals and hydrogen peroxide in *B. bassiana* mycelia following exposure to pyrethroids (Table 5).

**Table 5:** Percentage of hyphae containing superoxide anion radicals and hydrogen peroxide in *B. bassiana* after incubation with λ-cyhalothrin, α-cypermethrin, and deltamethrin.

		Control	Pyrethroids (100 mg L^-1^)		
			λ-cyhalothrin	α-cypermethrin	Deltamethrin
O_2_^·−^	24 h	1.76 ± 1.26	1.93 ± 1.02	4.09 ± 2.42*	2.37 ± 0.74
	36 h	0.55 ± 0.08	4.73 ± 0.82**	10.97 ± 4.15**	7.34 ± 1.44**
	48 h	1.51 ± 1.02	7.87 ± 3.25*	18.62 ± 3.22**	22.80 ± 5.31**
H_2_O_2_	24 h	nd	nd	nd	nd
	36 h	0.14 ± 0.00	0.22 ± 0.06	0.63 ± 0.31**	0.37 ± 0.12
	48 h	0.33 ± 0.26	3.80 ± 0.59**	4.64 ± 2.00**	3.28 ± 0.99**

Data are means ± SD; all samples were prepared in triplicate, and the experiments were repeated twice. Results were tested by one-way ANOVA; significance: **P  < 0.01, *P < 0.05. nd - not detected.

At the beginning of the experiment (24-h incubation), a slight increase in the level of superoxide anion radicals was observed only in the samples cultivated with α -cypermethrin, while H_2_O_2_ was not detected in the biomass. It has been found that under stress conditions, superoxide anion radicals appear first and are then converted by superoxide dismutase to hydrogen peroxide. In the present study, prolonging the incubation time resulted in a significant increase in the ROS levels. In the 36-h biomass, the amount of superoxide anion radicals increased by eightfold, almost 20-fold and 13-fold in the presence of λ-cyhalothrin (*P* = 0.008298), α-cypermethrin (*P* = 0.000161), and deltamethrin (*P* = 0.000194), respectively. Similarly, in 48-h cultures, approximately fivefold (*P* = 0.011729), 12-fold (*P* = 0.000175), and 15-fold (*P* = 0.000175) increase in the level of O_2_^·–^ were noted. A statistically significant increase in hydrogen peroxide by 11-fold, 14-fold, and ninefold was detected at 48 h of incubation in the samples exposed to λ-cyhalothrin (*P* = 0.000231), α-cypermethrin (*P* = 0.000177), and deltamethrin (*P* = 0.000585), respectively. These results clearly highlight that oxidative stress was induced in *B. bassiana* after the exposure to pyrethroids*.* Oxidative stress may have been caused by the entry and accumulation of pyrethroids in mycelium, which, as mentioned earlier, may be correlated with the increase in membrane permeability. Furthermore, the observed decrease of TAGs in the presence of pyrethroids may influence the generation of free radicals, the level of which may also depend on the amount of TAGs in cells.

Due to their lifestyle, entomopathogenic fungi may often experience oxidative stress by different factors, including exposure to insect host defense such as production of ROS^37^. Therefore, they are characterized by extremely flexible metabolism which enables them to grow under various environmental conditions. The results of our work confirmed that pyrethroids might be one of the harmful factors, which is in line with the observation made so far in fish and rats that oxidative stress is induced by these substances^38,39^.

### Effect of pyrethroids on secondary metabolite production by *Beauveria* species

**Figure 3:**
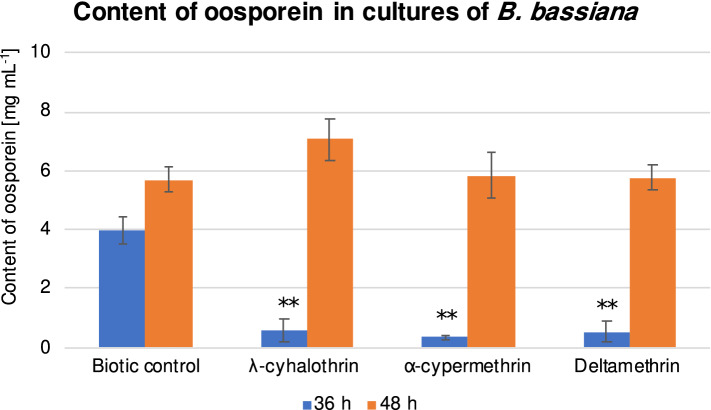
Content of oosporein [mg mL^−1^] in *B. bassiana* after 36 and 48 h of cultivation with λ-cyhalothrin, α-cypermethrin, and deltamethrin at a concentration of 100 mg L^−1^. All samples were prepared in triplicate, and the experiments were repeated twice. Results were tested by one-way ANOVA; significance: **P < 0.01, *P < 0.05.

Oosporein, a secondary metabolite belonging to the octadepsipeptide group, exhibits insecticidal, antiviral, and antibacterial effects^40^. It is an important metabolite of entomopathogenic fungi and stimulates infection in insects^7^. In our study, a lack of oosporein production was observed in the 24-h culture and the presence of pyrethroids was found to inhibit the pigment production even in the later period of incubation. In 36-h cultures, the content of oosporein was decreased in the samples exposed to pyrethroids than in the biotic control (λ-cyhalothrin: 6.7-fold (*P* = 0.000254), α-cypermethrin: 11.3-fold (*P* = 0.000242), deltamethrin: 7.5-fold (*P* = 0.000250)) (Fig. 3). In the 48-h culture, a slight increase in pigment content was observed in the samples with all tested insecticides, but these differences were not statistically significant (Fig. 3).

In general, microorganisms produce secondary metabolites to protect them against both biotic and abiotic stress^41^. The influence of toxic compounds on the production of these metabolites has been poorly understood, and so far only the inhibition of destruxins production by *Metarhizium* sp. by acetamiprid has been proved^42^. Temporary inhibition of oosporein production in the presence of pyrethroids indicates a harmful effect of these substances on entomopathogenic fungi and could be an effect of both slight growth inhibition and induction of oxidative stress, which could reduce the infectious potential of the fungi and thus adversely affect their behavior in the environment.

## Conclusions

Entomopathogenic fungi and pyrethroids do not only coexist in the environment but can also be applied together in pest control, and thus it seems highly important to understand the relationship between them. The findings of our study support the hypothesis that pyrethroids influence the metabolism of *B. bassiana* cells, especially the membrane lipid profile and permeability. By increasing the membrane permeability, pyrethroids gain access into the fungal cells and accumulate within them, leading to the formation of free radicals and reduction of sodium content. Intriguingly, these pesticides were found to cause a decrease in the level of sodium and temporarily lower the amount of the secondary metabolite oosporein, which suggests that their influence on cellular metabolism is very complex. For effective application of these two plant protection agents in pest control, understanding the mechanisms by which pyrethroids affect the metabolism of entomopathogenic fungi is critical, but the obtained results suggest that further research using tools such as proteomics or metabolomics is needed to fully uncover these phenomena.

## Supplementary Information

Supplementary Information.
