# Supplementary material for: Lipidomic response of the entomopathogenic fungus Beauveria bassiana to pyrethroids
Source: Sci Rep. 2021 Oct 29;11:21319. doi: 10.1038/s41598-021-00702-y (PMC8556296; doi:10.1038/s41598-021-00702-y)
Supplement: Supplementary file 1 — Supplementary Information. [file 41598_2021_702_MOESM1_ESM.pdf]

Lipidomic response of the entomopathogenic fungus *Beauveria bassiana* to pyrethroids

Anna Litwin[1], Przemysław Bernat[1], Monika Nowak[1], Mirosława Słaba[1], Sylwia Różalska[1]*

[1]Department of Industrial Microbiology and Biotechnology, Institute of Microbiology, Biotechnology and Immunology, Faculty of Biology and Environmental Protection, University of Lodz

*sylwia.rozalska@biol.uni.lodz.pl

Supplementary Table S1. Dry biomass (g L^{-1}) of *B. bassiana* after 36 and 48 h of cultivation with λ-cyhalothrin, α-cypermethrin, and deltamethrin.

	Pyrethroids (mg L^{-1})									
		λ –cyhalothrin			α –cypermethrin			Deltamethrin		
	Control	5	50	100	5	50	100	5	50	100
36 h	1.67 ± 0.14	1.75 ± 0.01	1.20 ± 0.23**	1.25 ± 0.03**	1.77 ± 0.01	1.26 ± 0.16**	1.34 ± 0.02**	1.71 ± 0.15	1.42 ± 0.07**	1.31 ± 0.05**
48 h	3.78 ± 0.21	3.93 ± 0.04	3.67 ± 0.06	3.19 ± 0.08**	4.03 ± 0.17	3.76 ± 0.11	3.29 ± 0.71*	3.89 ± 0.11	3.66 ± 0.09	3.56 ± 0.14

Data are means ± SD; all samples were prepared in triplicate, and the experiments were repeated twice. Results were tested by one-way ANOVA; significance: ** – $p < 0.01$, * – $p < 0.05$.

Supplementary Table S2. Number of blastospores (expressed as 10^7) in *B. bassiana* cultures supplemented with pyrethroids after 24 and 48 h of incubation.

	Pyrethroids (mg L^{-1})									
		λ -cyhalothrin			α -cypermethrin			Deltamethrin		
	Control	5	50	100	5	50	100	5	50	100
24 h	1.045 ± 0.46	0.989 ± 0.27	0.630 ± 0.22**	0.295 ± 0.10**	1.054 ± 0.36	0.692 ± 0.4**	0.322 ± 0.15**	1.008 ± 0.46	0.682 ± 0.17**	0.264 ± 0.10**
48h	7.453 ± 1.39	8.134 ± 2.61	1.597 ± 0.40**	0.856 ± 0.26**	7.55 ± 0.72	6.489 ± 1.58	4.436 ± 1.24**	7.768 ± 2.73	6.089 ± 0.84**	3.922 ± 0.90**

Data are means ± SD; all samples were prepared in triplicate, and the experiments were repeated twice. Results were tested by one-way ANOVA; significance: ** – $p < 0.01$, * – $p < 0.05$.

Supplementary Table S3. Phospholipid species composition determined in the *B. bassiana* cells after 48 h of cultivation with λ-cyhalothrin, α-cypermethrin, and deltamethrin.

Phospholipid speciess relative abundance (%)	Pyrethroids (mg L^{-1})			
	Control	λ –cyhalothrin	α –cypermethrin	Deltamethrin
PA 16:0 18:2	0.03 ± 0.02	0.03 ± 0.01	0.02 ± 0.01	0.02 ± 0.01
PA 18:2 18:2	0.05 ± 0.01	0.03 ± 0.02	0.07 ± 0.02	0.04 ± 0.01
PC 16:0 18:2	3.07 ± 0.06	3.62 ± 0.22**	4.16 ± 0.11**	3.92 ± 0.03**
PC 18:3 18:3	0.11 ± 0.01	0.16 ± 0.02**	0.18 ± 0.02**	0.17 ± 0.02**
PC 18:3 18:2	2.23 ± 0.04	2.45 ± 0.21*	2.68 ± 0.13**	2.53 ± 0.08**
PC 18:3 18:1	0.48 ± 0.10	0.49 ± 0.04	0.38 ± 0.03*	0.47 ± 0.04
PC 18:2 18:2	29.88 ± 0.25	34.49 ± 0.64**	33.85 ± 0.43**	33.15 ± 0.39**
PC 18:2 18:1	12.79 ± 0.72	11.58 ± 0.68**	10.61 ± 0.47**	11.67 ± 0.71*
PC 18:1 18:1	2.52 ± 0.36	1.87 ± 0.25**	1.52 ± 0.05**	1.84 ± 0.15**
PE 16:0 18:2	21.34 ± 1.11	18.04 ± 1.54**	18.62 ± 2.44*	18.89 ± 1.30*
PE 18:3 18:2	0.75 ± 0.05	0.80 ± 0.14	1.00 ± 0.06**	0.91 ± 0.04*
PE 18:2 18:2	8.19 ± 0.64	8.78 ± 0.35	11.34 ± 0.79**	10.35 ± 0.74**
PE 18:2 18:1	3.32 ± 0.14	3.55 ± 0.46	3.24 ± 0.33	3.28 ± 0.31
PE 18:1 18:1	2.61 ± 0.23	2.33 ± 0.19*	1.68 ± 0.14**	2.04 ± 0.14**
PE 16:0 18:1	7.22 ± 0.35	5.26 ± 0.41**	4.07 ± 0.25**	4.88 ± 0.48**
PE 18:2 18:0	2.05 ± 0.08	2.07 ± 0.08	2.23 ± 0.08	1.85 ± 0.28
PI 16:0 18:2	1.68 ± 0.06	2.02 ± 0.15**	1.90 ± 0.29	1.83 ± 0.10
PI 18:1 18:3	0.01 ± 0.00	0.01 ± 0.00	0.01 ± 0.00	0.01 ± 0.00
PI 18:2 18:2	0.94 ± 0.01	1.59 ± 0.31**	1.56 ± 0.17**	1.45 ± 0.09**
PI 18:1 18:2	0.16 ± 0.01	0.24 ± 0.03**	0.21 ± 0.02**	0.21 ± 0.01**
PS 16:0 18:2	0.31 ± 0.01	0.33 ± 0.02	0.32 ± 0.03	0.32 ± 0.01
PS 18:2 18:2	0.24 ± 0.08	0.24 ± 0.09	0.30 ± 0.02	0.13 ± 0.00
PS 18:1 18:2	0.03 ± 0.01	0.04 ± 0.01	0.04 ± 0.00	0.04 ± 0.01

Data are means ± SD; all samples were prepared in triplicate, and the experiments were repeated twice. Results were tested by one-way ANOVA; significance: ** – $p < 0.01$, * – $p < 0.05$.

Supplementary Table S4. Percentage content of tri- and diacylglycerols and TAG/DAG ratio in the *B. bassiana* cells after 48 h of cultivation with λ-cyhalothrin, α-cypermethrin, and deltamethrin.

	Pyrethroids (mg L^{-1})			
	Control	λ –cyhalothrin	α –cypermethrin	Deltamethrin
TAG 48:0	3.54 ± 0.27	10.08 ± 0.29**	11.65 ± 0.21**	4.05 ± 0.68
TAG 50:0	1.16 ± 0.04	2.80 ± 0.12**	3.39 ± 0.31**	1.21 ± 0.01
TAG 50:1	6.21 ± 0.49	5.70 ± 0.22	6.60 ± 0.01	6.25 ± 0.58
TAG 52:1	3.23 ± 0.05	7.14 ± 0.46**	8.45 ± 0.84**	3.52 ± 0.41
TAG 52:2	14.37 ± 0.70	17.30 ± 0.17**	15.24 ± 0.75	14.51 ± 0.46
TAG 52:3	10.21 ± 0.17	10.74 ± 0.87	8.29 ± 0.08**	10.46 ± 0.01
TAG 52:4	8.15 ± 0.51	7.42 ± 0.11*	7.31 ± 0.39*	8.06 ± 0.17
TAG 54:0	1.46 ± 0.06	1.55 ± 0.05	1.51 ± 0.15	1.91 ± 0.06**
TAG 54:1	3.63 ± 0.11	1.35 ± 0.35**	1.10 ± 0.06**	3.63 ± 0.20
TAG 54:2	5.07 ± 0.33	2.16 ± 0.01**	1.98 ± 0.17**	3.44 ± 0.57**
TAG 54:3	16.17 ± 0.97	13.68 ± 0.84**	12.44 ± 0.36**	16.98 ± 0.64
TAG 54:4	16.27 ± 0.93	11.25 ± 0.12**	13.43 ± 1.18**	15.29 ± 0.68
TAG 54:6	10.52 ± 0.26	8.83 ± 0.10**	8.61 ± 0.44**	10.70 ± 0.53
DAG 32:0	3.29 ± 0.26	9.96 ± 1.44**	5.43 ± 0.47**	1.72 ± 0.01
DAG34:0	2.08 ± 0.01	2.97 ± 0.48**	2.63 ± 0.22	2.46 ± 0.25
DAG34:1	1.75 ± 0.04	2.05 ± 0.56	2.16 ± 0.31	1.98 ± 0.19
DAG34:2	12.32 ± 0.37	15.00 ± 0.11**	16.99 ± 0.01**	12.30 ± 1.04
DAG36	3.54 ± 0.42	2.74 ± 1.34	4.26 ± 1.43	4.35 ± 1.21
DAG36:1	34.12 ± 1.59	21.60 ± 3.90**	23.00 ± 1.44**	39.17 ± 0.26*
DAG36:2	36.93 ± 2.67	40.07 ± 4.58	40.36 ± 1.37	33.66 ± 1.28
DAG36:3	4.97 ± 0.71	4.64 ± 1.00	4.32 ± 0.77	3.57 ± 0.87
DAG36:4	1.00 ± 0.12	0.96 ± 0.62	0.84 ± 0.35	0.80 ± 0.26
TAG/DAG	0.41 ± 0.00	0.41 ± 0.02	0.24 ± 0.02**	0.37 ± 0.02

Data are means ± SD; all samples were prepared in triplicate, and the experiments were repeated twice. Results were tested by one-way ANOVA; significance: ** – $p < 0.01$, * – $p < 0.05$.

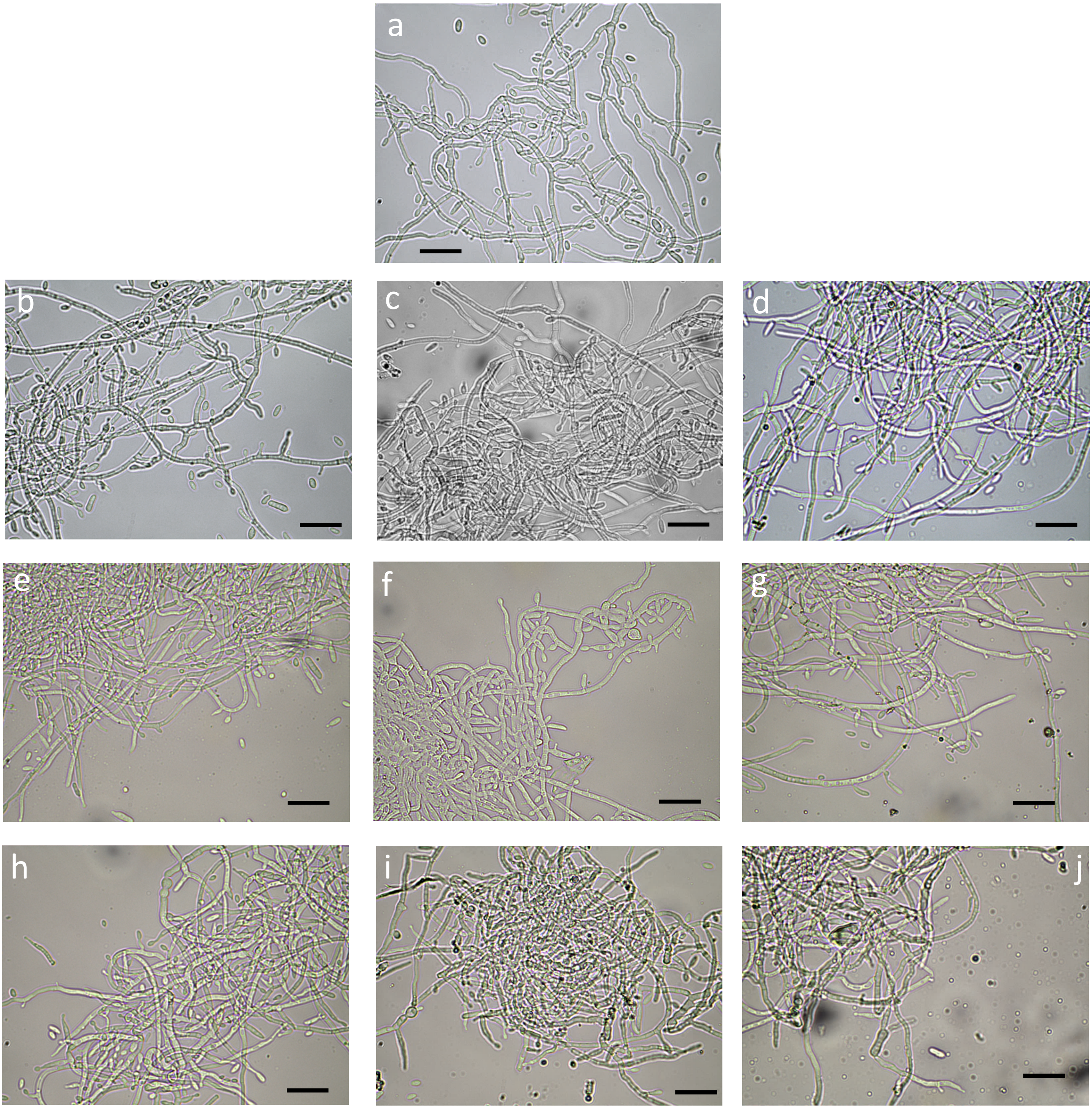

Supplementary Figure S1. Morphology of *B. bassiana* in 24 h cultures supplemented with pyrethroids. Control without toxic substances (a); λ-cyhalothrin at concentrations of 5, 50 and 100 mg L^{-1} (b-d, respectively), α-cypermethrin at concentrations of 5, 50 and 100 mg L^{-1} (e-g, respectively) and deltamethrin at concentrations of 5, 50 and 100 mg L^{-1} (h-j, respectively).
The scale bar represents 20 μm.

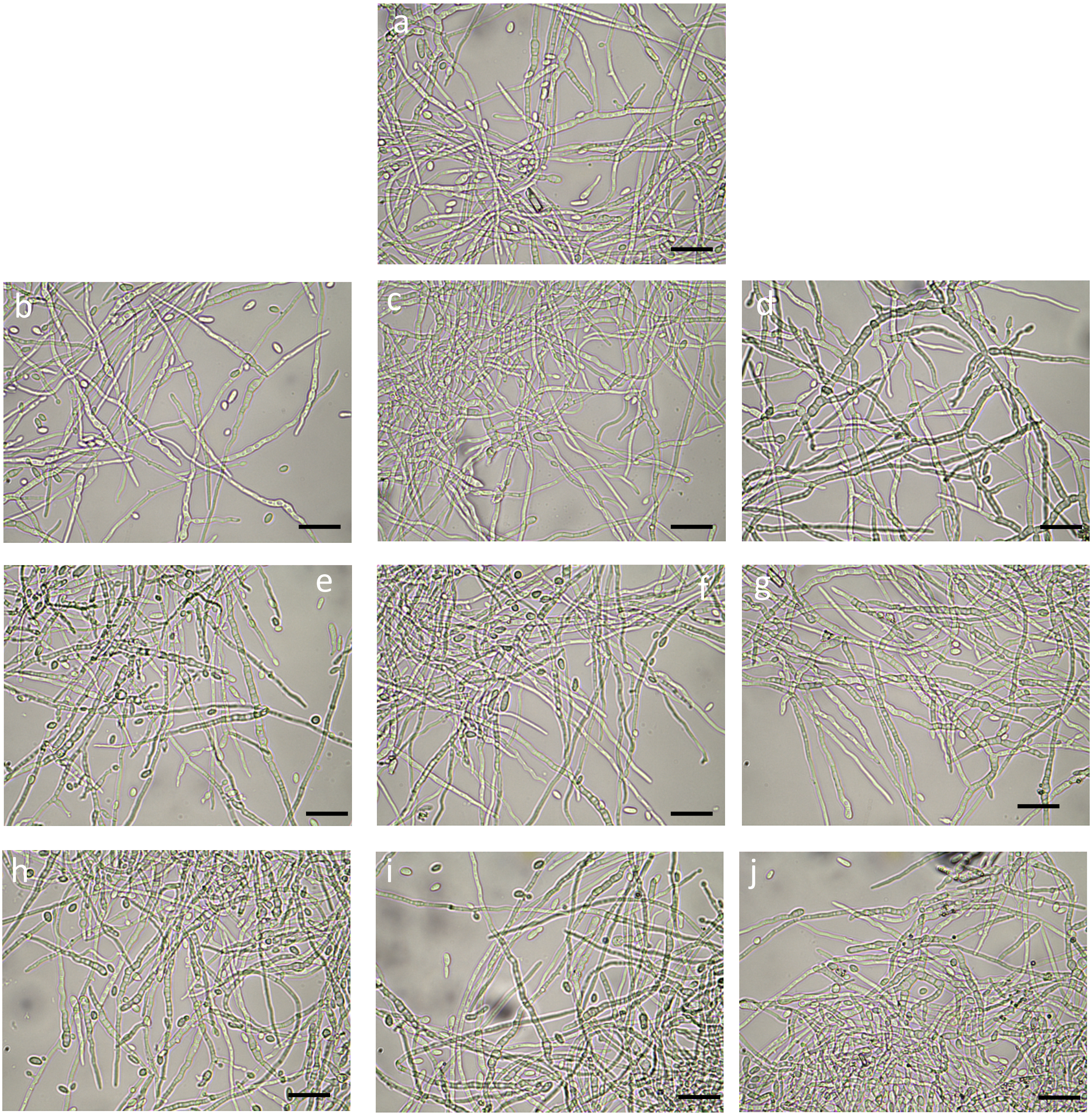

Supplementary Figure S2. Morphology of *B. bassiana* in 48 h cultures supplemented with pyrethroids. Control without toxic substances (a); λ-cyhalothrin at concentrations of 5, 50 and 100 mg L^{-1} (b-d, respectively), α-cypermethrin at concentrations of 5, 50 and 100 mg L^{-1} (e-g, respectively) and deltamethrin at concentrations of 5, 50 and 100 mg L^{-1} (h-j, respectively).
The scale bar represents 20 μm.
